# Genome Wide Association Study for Drought, Aflatoxin Resistance, and Important Agronomic Traits of Maize Hybrids in the Sub-Tropics

**DOI:** 10.1371/journal.pone.0117737

**Published:** 2015-02-25

**Authors:** Ivan D. Barrero Farfan, Gerald N. De La Fuente, Seth C. Murray, Thomas Isakeit, Pei-Cheng Huang, Marilyn Warburton, Paul Williams, Gary L. Windham, Mike Kolomiets

**Affiliations:** 1 Department of Soil and Crop Sciences, Texas A&M University, College Station, Texas, United States of America; 2 Department of Plant Pathology, Texas A&M University, College Station, Texas, United States of America; 3 USDA ARS Corn Host Plant Resistance Research Unit, Mississippi State, Mississippi, United States of America; University of Guelph, CANADA

## Abstract

The primary maize (*Zea mays* L.) production areas are in temperate regions throughout the world and this is where most maize breeding is focused. Important but lower yielding maize growing regions such as the sub-tropics experience unique challenges, the greatest of which are drought stress and aflatoxin contamination. Here we used a diversity panel consisting of 346 maize inbred lines originating in temperate, sub-tropical and tropical areas testcrossed to stiff-stalk line Tx714 to investigate these traits. Testcross hybrids were evaluated under irrigated and non-irrigated trials for yield, plant height, ear height, days to anthesis, days to silking and other agronomic traits. Irrigated trials were also inoculated with *Aspergillus flavus* and evaluated for aflatoxin content. Diverse maize testcrosses out-yielded commercial checks in most trials, which indicated the potential for genetic diversity to improve sub-tropical breeding programs. To identify genomic regions associated with yield, aflatoxin resistance and other important agronomic traits, a genome wide association analysis was performed. Using 60,000 SNPs, this study found 10 quantitative trait variants for grain yield, plant and ear height, and flowering time after stringent multiple test corrections, and after fitting different models. Three of these variants explained 5–10% of the variation in grain yield under both water conditions. Multiple identified SNPs co-localized with previously reported QTL, which narrows the possible location of causal polymorphisms. Novel significant SNPs were also identified. This study demonstrated the potential to use genome wide association studies to identify major variants of quantitative and complex traits such as yield under drought that are still segregating between elite inbred lines.

## Introduction

Maize (*Zea mays L*.) is one of the three most important crops of the world along with rice (*Oryza sativa*) and wheat (*Triticum spp*.). World production in 2011 was 883 million ton (http://faostat.fao.org [verified 6 May 2013]). Maize is the most important crop in the United States with a production of 301 million tons, and an estimated market value of 77.4 billion U.S. dollars in 2012 (National Agricultural Statistical Service [NASS] 2013). Globally, the most important and highest yielding production areas are in the temperate Midwestern U.S. and other temperate regions throughout the world, which is where the majority of investment in maize breeding is focused [1–3]. Important but lower yielding maize growing regions, such as the sub-tropics, experience unique challenges. Sub-tropical production zones are hotter and drier, and two of the greatest challenges for maize production in these zones are drought stress and aflatoxin contamination [4–12]. Sub-tropical maize production in the U.S. Southern states accounts for approximately 9% of U.S. production [13]. The largest producer in the Southern states is Texas, where summers are hot and dry. There is a strong inter-annual precipitation variation across the state and severe drought episodes have occurred in the last ten years [14]. These challenges may become increasingly common in temperate regions under a changing climate. Texas provides ideal environmental conditions to conduct research in drought tolerance and aflatoxin contamination that major maize production regions could experience in the future.

Improving drought tolerance is important because agriculture is the major user of surface and ground water in the U.S. It has been estimated that water usage in agriculture for the Western states accounts for up to 90% of the total water used in these states [15]. The use restrictions and competition for water by growing urban areas will make drought stress even more common in irrigated agriculture. Drought episodes are likely to increase in the Midwestern U.S. because of a stronger inter-annual variation in precipitation and temperature attributable to a changing climate [16,17]. Drought tolerance is difficult to quantify and improve, as it is clearly quantitative and complex, and regulated by thousands of genes [18–20].The impact of drought stress depends on the severity, timing and length of the stress. Maize is most sensitive to drought stress during flowering and grain fill stages [18,21,22]. Drought stress in maize causes reduction in plant height, leaf rolling, early senescence, asynchronous flowering, kernel abortion and barren plants without ears [18,21,22].

Drought episodes and other stresses such as heat are often followed by pre-harvest aflatoxin contamination. Aflatoxin is a carcinogenic mycotoxin, produced by the soil borne fungus *Aspergillus flavus*, which thrives under hot and dry conditions in pre-harvest maize. Aflatoxin is federally regulated at 20 ng g^−1^ for human consumption, and is believed to cause over $200 million dollars of economic losses in the Southern U.S. each year [4,12,23,24]. Aflatoxin susceptibility in plants is a highly complex trait and no complete source of resistance is known for maize [7,25]. Adding to the complexity of this pathogen, both colonization and aflatoxin production appears to have strong host-by-pathogen interactions [26–29]. As a consequence, breeding for aflatoxin resistance is a complex challenge. Despite this complexity a number of breeding lines and germplasm with improved aflatoxin resistance have been released. The lines Mp313E, Mp715, and Mp717 [30,31] were derived from the tropical maize race Tuxpeño after several cycles of selection. The maize inbred lines Tx736, Tx739, Tx740, and Tx772 [7,25,32] were selected using the pedigree method from Argentinean and Bolivian lines. Other sources of resistance such as the line GT603 were selected from temperate elite hybrids produced in the 1970s [33]. This diverse germplasm has already been used in linkage mapping studies to identify the quantitative trait loci (QTL) responsible for conferring resistance [11,23,34–39]. Relatively large QTL for aflatoxin resistance have been reported on chromosomes one, three, four, five, and nine [25,34,39,40] and there has often been little consistency between germplasm and environments. These findings give evidence that diverse germplasm has been the primary and best source for reducing ear rot diseases, and more importantly that decreased susceptibility is indeed heritable [25,30,31,41]. Diverse tropical maize germplasm has also demonstrated the potential to out-yield commercial hybrids in some environments. This germplasm also can exhibit improved drought tolerance and other unique traits unavailable in commercial temperate hybrids when testcrossed to elite temperate lines [42–48]. However, tropical germplasm also may have many undesirable traits, such as delayed flowering time/ photoperiod sensitivity and dry down, lower yield in some cases, poor stalks, and disruption of heterotic patterns which makes it challenging to use in temperate breeding programs [45,46,48].

Diverse germplasm must be further investigated to identify new sources of genetic variation for drought stress and aflatoxin contamination outside of the elite Midwestern germplasm such as the so called exPVP’s [45]. One source of diverse germplasm previously characterized for the maize research community is the 282 Goodman maize association panel [43,49,50]. The 282 maize association panel includes several lines that originated from the dent maize commercial lines in the 1970s and 1980s [51], and multiple North Carolina and CIMMYT lines of tropical origin. Another diversity source includes the lines that have been bred in the Southern States by public programs, including those selected and released for aflatoxin resistance. The identification of favourable alleles in these sets of germplasm can allow targeted genetic improvement of current germplasm, and the incorporation of those alleles into elite material. In addition, diverse germplasm with differences in phenotype facilitates linkage mapping and association studies [52]. Multiple bi-parental linkage QTL mapping studies on aflatoxin and drought have found many loci which tend to be specific to a narrow set of genetic backgrounds, but not maize as whole. In contrast, genome wide association studies (GWAS) allow evaluating multiple alleles in multiple genetic backgrounds at once. GWAS also captures many more effective recombination events than traditional linkage populations, and therefore has the potential to increase QTL resolution when the assembled germplasm panels have low linkage disequilibrium (LD).

Complex trait analysis can benefit from a number of statistical adjustments that account for data dependencies such as field spatial variation or blocking effects. A number of spatial adjustment methods have been developed, which can decrease the micro-environment non-genetic noise in an experiment, making the results more robust and powerful [53–57]. These spatial adjustment procedures are particularly appropriate in large field trials with heterogeneous soil or furrow irrigation patterns. In addition, the modelling of the genetic by environmental interaction (G x E) can allow detection of QTL specific for certain environments [58,59]. These more complex models maximize the amount of genetic variation explained and model the genetic variance and correlation across environments. As a result, the power of the data is increased, while reducing false positives. Similarly, statistical methods to account for genetic population structure and relatedness have long been used in association mapping to account for spurious associations and provide a more conservative analysis [60]. Finally, a number of multiple testing corrections procedures have been developed, each with various advantages and drawbacks [61–63]. However, despite the various statistical analyses corrections (spatial adjustment, modelling G x E, genetic relatedness, and multiple testing), it has been determined that some associations are still subject to false positives (and negatives) which may only be identified retrospectively [64]. In past work, we have found that attempting multiple analysis methods and ensuring consistency between results suggests the most robust candidates for further analysis [65,66], this can come at the expense of clarity in presentation.

This study used 346 lines from across two diversity panels testcrossed to Tx714, which is a high yielding but aflatoxin susceptible line that is closely related to B73, to evaluate alleles from diverse germplasm in Southern US environments. Specifically, the goals of this study were: 1) to identify diverse lines to improve yield potential, aflatoxin resistance, and agronomic abilities in hybrid combination; and 2) to identify genomic regions that confer these phenotypes in hybrids grown in Southern sub-tropical environments using a genome wide association study (GWAS).

## Materials and Methods

### Phenotypic data collection

An association mapping panel comprised of subsets of the USDA Goodman maize association panel (302 lines, [49]) and the diverse Southern subtropical focused Williams/Warburton panel (300 lines, [67]) was assembled for a total of 400 lines [68]. Of the 400 lines, 346 produced sufficient seed in testcrosses to Tx714 [69] in a summer and fall nursery in College Station, TX and Weslaco, TX, respectively in both 2010 and 2011. The hybrids were evaluated in two separate replicates in a randomized complete block design (RCBD) under irrigated and non-irrigated treatments with commercial checks randomly assigned in the field. Standard production agronomics were followed to provide furrow irrigation to the irrigated trials as needed to support crop growth. Only the well-watered trials were inoculated with *A*. *flavus*. A modified colonized kernel technique was used where autoclaved maize kernels were inoculated with *A*. *flavus* spores from isolate NRRL 3357 [70] and then incubated 24 to 36 hours to promote *A*. *flavus* growth and sporulation. Colonized kernels were manually spread on the soil surface between treatment rows when the maize hybrids reached mid-silk stage [4,71]. The testcross hybrids were grown in College Station (CS) in 2011 (CS11) and 2012 (CS12) at the Texas A&M AgriLife Research Farm in one row plots 7.92 meters long and 76.2 centimetres wide, and measured for all reported traits as explained below. The target plant density was 75,000 plants/ha and the soil type was a Ships clay. Combination of year and treatment were designated trials and the following coding system was adopted in this research: The irrigated trials were inoculated with *A*. *flavus* and coded well watered (WW) and the non-irrigated trials were coded as water stress (WS). The water stress was most severe in 2011, especially during flowering and grain filling. Water stress was less severe at these times in 2012 because of moderate temperatures and timely rainfall. An additional well watered location was evaluated in two replicates using a RCBD at Mississippi State, MS in 2012 (MS12) only for aflatoxin accumulation and days to silk. Hybrids in this location were inoculated using the side needle method [71–73].

Plant height was measured from the ground to top of the tassel; ear height was measured to the bottom ear node. Days to silk and days to anthesis were measured as the number of days, at which 50% of the plants in a plot showed silks or pollen shed respectively. Anthesis silking interval (ASI) was calculated using the difference between days to silk and days to anthesis. Because the severe drought in Texas in 2011 substantially reduced ear size, the CS11-WS trial was completely hand-harvested. In contrast, for the CS12-WS, CS11-WW and CS12-WW, 10 ears were hand harvested skipping the first five plants in the plot, and then hand harvesting every other plant. The rest of the plot was harvested using a John Deere 3300 combine with a HM-1000B Grain Gauge (Juniper Systems Inc., Logan, Utah) from which plot weight, moisture and test weight were obtained. For the MS12-WW trial only the 10 inoculated ears were hand harvested and processed for aflatoxin content. Hand harvested ears from each hybrid in CS were photographed and phenotyped for disease, percentage of kernel abortion and pollination. 500-kernel weight was determined after shelling. All yields were adjusted to 15.5% moisture as determined from the combine at harvest or for hand harvested ears after shelling using a Dickey-John mini GAC plus portable moisture tester. Moisture was expressed as percentage of weight. Aflatoxin content was determined by the Vicam Aflatest fluorometer (Vicam, Watertown, MA) following standard procedures [7,37,39]. Aflatoxin values were transformed using the transformation (Log10 [aflatoxin + 10]) to improve normality and constant variance and back-transformed. Raw phenotypic data can be found in S1 Table.

### Phenotypic analysis for the GWAS study

The different hybrids within each treatment and each location were laid out using a randomized complete block (RCBD) design with commercial checks randomly assigned to different plots in the trial. Four commercial checks (check 1: BH9014 GENVT3P, check 2: GA26V21 GENVT3P, check 3: GA28V81 GENVT3P, check 4: DKC-805 GENVT3P) were replicated several times in the field and were used to adjust for field spatial variation and to estimate the residual variance. These commercial checks were excluded for estimating variance components and Best Linear Unbiased Predictors (BLUPs) for the GWAS. A combined multi-environment trial (MET) analysis was performed considering three different models: 1) a randomized complete block design (RCBD) model, 2) a spatial model that used autoregressive terms for the row and column effects (AR1 x AR1), and 3) to model the G x E, an unstructured genetic variance-covariance matrix model (VCOV). The unstructured model assumes that every location has its own genetic variance and that the genetic covariance between locations is different. The likelihood ratio test was used to compare between the three different models for each trait, but not all models could be estimated for all traits because of differences in the heritability estimates for the different traits.

An RCBD model was first fit to the data. The phenotypic observation *y*_*ijk*_ on hybrid *i* in replicate *j* of trial *k* was modelled as:

$$Y_{ijk}\;=\;\mu \;+\;e_{k}\;+\;g_{i}\;+\;{\left(r/e\right)}_{jk}\;+\;{\left(g*e\right)}_{ik}\;+\varepsilon_{ijk}$$ (1)

where, *μ* is the grand mean; *e*_*k*_ is the fixed effect of trial *k; g*_*i*_ is the random effect of hybrid *i* and is ∼ NID (0, *σ²*_*g*_), i = 1, …,g; *(r/e)*_*jk*_ is the random effect of replication *j* nested in environment *k* and is ∼ NID (0, *σ²*_*r*_), *r = 1*,*2*; (g*e)_*ik*_ is the random effect of the interaction between hybrid *i* and trial *k* and is NID (0, σ²_*ge*_), and *ε*_*ijk*_ is the random residual effect for hybrid *i* in the replication *j* of trial *k* and is NID (0, *σ²*_*ε*_). For the second model the phenotypic observation *y*_*ijk*_ on hybrid *i* in *k* was modelled as:

$$Y_{ijk}\;=\;\mu \;+\;e_{k}\;+\;g_{i}\;+\;{\left(g*e\right)}_{ik}\;+\varepsilon_{ijk}$$ (2)

where, *μ* is the grand mean; *e*_*k*_ is the fixed effect of trial *k; g*_*i*_ is the random effect of hybrid *i* and is ∼ NID (0, *σ²*_*g*_), i = 1, …,g; (g*e)_*ik*_ is the random effect of the interaction between hybrid *i* and trial *k* and is NID (0, σ²_*ge*_), and *ε*_*ijk*_ is the random residual effect for hybrid *i* in the replication *j* of trial *k* and is NID (0, *σ²*_*ε*_). *ε*_*ijk*_ was further expanded to fit two-dimensional autoregressive first order (AR1) x (AR1) terms for the row and column effects[53–57].

A third model assumes a genetic variance-covariance (VCOV) matrix based on an unstructured model for the random genetic effects where a specific variance was fit for each trial and a specific covariance was fit for each pair of trials. The phenotypic observation *Y*_*ijk*_ on hybrid *i* of trial *k* was modelled as:

$$Y_{ijk}\;=\;\mu \;+\;e_{k}\;+\;g_{i}\;+\;{\left(r/e\right)}_{jk}\;+\;{\left(gge\right)}_{ik}\;+\varepsilon_{ijk}$$ (3)

where, μ is the grand mean; e_k_ is the fixed effect of trial k; gge_i_ represents the hybrid main effect together with the genetic environmental interaction (GEI) for hybrid i in trial k. This model was enhanced by including the significant terms for autoregressive terms for row and column effects. Each of the three different models were fit using restricted maximal likelihood (REML) (Patterson and Thompson, 1975) in ASREML v3.0 [57]. JMP Pro V11.1.1 was used in testing for significance of yield and aflatoxin in hybrids, treated as fixed, using similar models. Heritability (h^2^) was calculated using the equation:

$$h^{2}\;=\;1-PEV/2\sigma_{g^{2}}$$ (4)

[55,74]. The predictor error variance (PEV) was calculated as square root of the average of the standard error for each hybrid prediction for a given trait. The standard error was obtained from the output in ASREML.

### Genetic diversity, population structure and estimation of kinship matrix

Genome-wide single nucleotide polymorphism (SNP) genotype data from 213 lines from the sub-tropical diverse panel was obtained from the USDA-ARS Corn Host Plant Resistance Research Unit (Mississippi State, MS) using the Genotype By Sequence (GBS) method [75]. All SNP locations are based on the maize genome version AGPV2. Genotype data for 133 lines from the temperate panel was extracted from the maize diversity panel of 282 inbred lines available in Panzea [49]. These genotypes were identical to Warburton et al. [67] with the additional lines obtained from the 282 association mapping panel [49]. SNPs with a minor allele frequency (MAF) greater than 25% and a low missing data rate (<7.5%) were extracted to perform the genetic diversity and structure analysis only (total 1999 SNPs). The genetic distance was calculated for Nei’s genetic distance [76] using the software PowerMarker. A principal coordinate analysis (PCoA) was then carried out using the prcomp function in R (R Development Core Team, 2013). Population structure was determined using the software Structure v2.93 [77]. The number of subpopulations was estimated from five independent runs having 5 x 10^5^ burn-in and sampling iterations, and the number of subpopulations was varied between 1 and 15. The ancestry model allowed for population admixture and correlated allele frequencies. The optimum *K* was estimated using the ad hoc statistic *∆K*, which is based on the rate of change in the log probability of the data between successive *K* values [78]. Based on the estimated K determined, a run of 5 x 10^6^ burn-in and sample iterations was used. A kinship coefficient estimation matrix was created using the VanRaden algorithm as implemented in the software GAPIT [79].

### Genome wide association study (GWAS)

For each trait analysed, up to four different phenotypic inputs were used for GWAS analysis to ensure that quantitative trait variants (QTVs) were robust across models, and to identify QTV’s for specific environments. The phenotypic observations used as input for the four GWAS analyses included 1) the entry mean for the different traits collected for all trials; 2) the BLUPs for the combined MET analysis in Eq. (1); 3) the BLUPs for the MET analysis in Eq. (2) including the row and column (AR1) x (AR1) effects for the spatial analysis; and 4) the BLUPs from the VCOV GEI analysis in Eq. (3). Population structure and relatedness were taken into account in the linear mixed model in all four analyses as described by Yu et al. [60]. The association mapping analysis was conducted using the compressed mixed linear mixed model (CMLM) and the P3D method, as implemented in GAPIT [79–81]. SNPs with a MAF< 0.05 were discarded for the association mapping analysis as they are prone to false positives [82]. Two common correction procedures were used to control for multiple testing, since each procedure has unique advantages and disadvantages [61,83]. First, the p-value was corrected for multiple testing using the modified method proposed by Gao et al. [62]. This approach calculates the M_eff_, which is the effective number of independent test to correct for multiple testing. M_eff_ is determined by estimating the pair-wise composite LD matrix [62,63,84]. The number of principal components (PCs) required explaining 99% of the variation in the dataset was used to calculate the Bonferroni correction. The M_eff_ calculated for this study was 49,030 independent tests, which is equivalent to 1.01 x 10^-6^ or 5.99 (-log10[p]). Second, the false discovery rate (FDR) was also used to correct for multiple testing for all the different analysis and traits in this study but can overcorrect, leading to type II error [85].

## Results and Discussion

### Variance component estimates and heritability within and across environments

The genetic variance in this experiment was very high. The heritability estimates for the WW and WS trials for grain yield ranged from 0.61 to 0.83 (Table 1). The lowest heritability in the WS trial was reported during the extreme drought of 2011. This result is expected since heritability estimates are expected to decrease under stressed trials [21,86–89]. Comparable heritability estimates for yield under drought stress trials have been reported previously [53,90]. Within each environment, heritability estimates for aflatoxin level ranged from 0.67 to 0.83 for both the transformed and raw aflatoxin level data (Table 1) demonstrating high statistically significant variation between genotypes. However, a lower heritability estimate of 0.59 across all environments was obtained (Table 2). Transformation of aflatoxin is a standard procedure used prior to QTL mapping [7,34,39], and it is needed because the data are often skewed. In this study, data transformation increased the heritability in some, and across all, environments (Table 2).

**Table 1:** Variance components and heritability estimates for each individual trial.

	Grain yield (ton/ha)		Aflatoxin (ng g−1)		Log10 [aflatoxin (ng g−1)+10]	
Effect	CS11-WW	CS11-WS	CS11-WW	MS11-WW	CS11-WW	MS11-WW
σ²_*g*_	299.9 ± 41.3	59.3 ± 16.6	233,845 ± 36,311.3	NA^a^	0.05 ± 0.009	NA^a^
σ²_*r*_	536.2 ± 385.8	20.5 ± 22.8	3,008.1 ± 4,359.6	NA^a^	0.001 ± 0.001	NA^a^
σ²_*ε*_	534.5 ± 28.9	187.1 ± 17.1	264,382 ± 22,235.6	NA^a^	0.08 ± 0.006	NA^a^
h²	0.73	0.61	0.81	NA^a^	0.77	NA^a^
	**CS12-WW**	**CS11-WS**	**CS12-WW**	**MS12-WW**	**CS12-WW**	**MS12-WW**
σ²_*g*_	767.9 ± 81.3	732.8 ± 83.9	296.7 ± 309	652,827 ± 87,276.3	0.07 ± 0.02	0.118 ± 0.02
σ²_*r*_	7.6 ± 10.7	28.6 ± 34	3,173. 8 ± 837.4	10,654.5 ± 18,058.5	0.004 ± 0.004	0.002 ± 0.004
σ²_*ε*_	703.6 ± 32.8	562.5 ± 50	11,612 ± 815.4	617,598 ± 51,338.2	0.2 ± 0.01	0.181 ± 0.015
h²	0.83	0.8	0.67	0.83	0.7	0.7

^a^ Data was not collected for this trait for this trial.

Estimates of the hybrid (σ²_*g*_), replicate (σ²_r_), error (σ²_*ε*_) variances and their respective standard errors for each trial for grain yield (ton/ha) and aflatoxin (ng g^−1^). The estimates were obtained using a linear mixed model that fit hybrids and replicate as a random effect and commercial checks as a fixed effect. CS11-WS College Station 2011 water stress, CS11-WW College Station 2011 well watered, MS11-WW Mississippi state 2011 well watered, CS12-WS College Station 2012 water stress, CS12-WW College Station 2012 well watered, MS12-WW Mississippi state 2012 well watered.

**Table 2:** Variance components and heritability estimates for all traits across all trials.

Effect	Days to anthesis	Days to silk	ASI	Plant height (cm)	Ear height (cm)	500-kernel weight (gr)	Grain yield (ton/ha)	Aflatoxin (ng g^−1^)	Log10 (aflatoxin (ng g^−1^))
σ²_*g*_	7.8 ± 0. 5	8.5 ± 0.5	0.3 ± 0.04	228.5 ± 19.2	107.3 ± 9.2	122.1 ± 11.5	0.08 ± 0.008	47465 ± 17197.5	0.04 ± 0.008
σ²_*ge*_	0.3 ± 0.03	0.4 ± 0.05	0.2 ± 0.03	32.2 ± 3.6	13.8 ± 2.1	42.5 ± 4.2	0.02 ± 0.003	238610 ± 24273.7	0.04 ± 0.008
σ²_*ε*_	0. 7 ± 0.02	0.9 ± 0.03	0.7 ± 0.02	91.7 ± 2.8	63.2 ± 1.9	94.2 ± 2.8	0.1 ± 0.003	273774 ± 12332.2	0.2 ± 0.007
h²	0.98	0.97	0.82	0.93	0.92	0.88	0.87	0.59	0.70

The variance components were estimated using the combined MET analysis in Eq. (1) without spatial adjustment for all the traits. These were derived from the hybrids (*g*), the interaction between the hybrid (*i*) and trial (*e*), and the experimental error (ε). Heritability was estimated using the method proposed by Cullis et al. [55], and Oakey et al. [74].

Across all environments and all reported traits, heritability estimates ranged from 0.59 for aflatoxin to 0.98 for days to anthesis (Table 2), based on the components of variance estimated using the MET model from Eq. (1). These heritability estimates are considerably higher than estimates reported in previous studies [7,39,72,91]. The differences observed in the heritability estimates for grain yield and aflatoxin level between this study and others are partially explained by the high genetic variation present in this study. Based on the heritability estimates observed, it was concluded that sufficient genetic variation was present in this panel to perform an association analysis.

### Spatial analysis for row column (AR1 x AR1), and G x E modelling using an unstructured genetic variance-covariance model

The number of hybrids investigated in the experiment resulted in large field tests. Spatial analysis has proven useful in identifying and reducing error in large field studies when checks are replicated throughout the field [54,56,92]. The analysis showed that field spatial variation was most important for for grain yield among traits reported (Table 3). CS11 was the location with the most field spatial variation for grain yield (data not shown). This would be expected with the extreme drought in 2011, as drought typically brings out the highest level of field variation. For other traits such as plant and ear height and 500 kernel weight field spatial variation was observed as well. In contrast, field spatial variation was not observed for aflatoxin contamination, however this could partially be due to the large variance (lower heritability) that would not allow row and column effects to be determined. Fitting these spatial models, when significant, partitions out error allowing more power for subsequent QTV detection.

**Table 3:** Comparisons of different models used in this analysis.

Model	Days to anthesis	Days to silk	ASI	Plant height (cm)	Ear height (cm)	Grain yield (ton/ha)	Aflatoxin
RCBD	-2636.5	-3090.2	-1654.1	-10173.4	-9427.1	1640.9	453.5
AR1 x AR1	NE	NE	-1651.1 (NS)	-9782.8***	-9324.5***	2055.8***	454.3 (NS)
VCOV	NE	NE	-1598.1***	-9624.3***	-9256.5***	2120.8***	490.8***

*** >0.001, NS (not significant), NE (Not estimated)

The log likelihood values are presented for the randomized complete block design (RCBD) model, the row column spatial analysis model (AR1 x AR1) and the unstructured genetic variance-covariance matrix model (VCOV). The likelihood ratio test was used to compare the log likelihood change between the different models. Not all models could be estimated (NE).

### Genetic correlation across different environments

A higher genetic correlation across environments would be expected in diverse material with a wide range in flowering, but the diversity in this study was moderated by the use of testcrosses. The modelling of the variance-covariance structure for GEI analysis using Eq. (2) [93] was greater than zero for all environment pairs (Table 4). This analysis showed the lowest genetic correlation for grain yield was 0.63, which correspond to the genetic correlation between CS11-WS and CS12-WW trials (Table 4). In contrast, the genetic correlation between other trials for grain yield ranged from 0.70 to 0.95. Based on these results, it was concluded that hybrid ranking between years and trials was consistent. This result is similar to other drought QTL studies in maize, sorghum and wheat [90,94,95]. The lowest genetic correlation for aflatoxin level was between the CS12-WW and MS12-WW trials (Table 5). This could in part be due to the different inoculation methods used which was confounded between these locations or other environmentally specific differences.

**Table 4:** Variance-covariance unstructured matrix for grain yield (ton/ha).

Environment	CS11-WS	CS11-WW	CS12-WS	CS12-WW
CS11_WS	0.06	0.76	0.70	0.63
CS11_WW	0.06	0.09	0.95	0.84
CS12_WS	0.07	0.11	0.15	0.94
CS12_WW	0.05	0.08	0.12	0.10

CS11-WS College Station 2011 water stress, CS11-WW College Station 2011 well watered, CS12-WS College Station 2012 water stress, and CS12-WW College Station 2012 well watered. Four trials were grown in College Station in 2011 and 2012. The diagonal represents the genetic variance for each trial. The elements off the diagonal in the lower half of the matrix are the specific genetic covariance per each pair of trials. The elements off the diagonal in the upper half of the matrix (shaded in gray) represent the specific genetic correlation for each pair of trials.

**Table 5:** Variance-covariance unstructured matrix for aflatoxin level (ng g^−1^) for the College Station 2011 and 2012 trials and Mississippi 2012 trial.

Environment	CS11-WW	CS12-WW	MS12-WW
CS11_WW	0.02	0.77	0.60
CS12_WW	0.04	0.10	0.46
MS12_WW	0.03	0.05	0.13

CS11-WW: College Station 2011 well watered, CS12-WW College Station 2012 well watered, MS12-WW Mississippi state 2012 well watered. The diagonal represent the genetic variance for each trial. The elements off the diagonal in the lower half of the matrix are the specific genetic covariance for each pair of trials. The elements off the diagonal in the upper half of the matrix represent the specific genetic correlation for each pair of trials.

### Best performing hybrids

There were multiple hybrids that performed statistically as well as, or better than, the elite commercial checks used in this study for grain yield (Table 6). This was evidence that diverse inbred lines bred in the test environment have the potential to out-yield elite commercial hybrids under water stress, even when combined with an older public tester. Under the severe drought that was experienced in Texas 2011, none of the commercial checks was among the fifteen highest yielding hybrids but three commercial checks were not significantly different from this group. In the CS11-WW and CS12-WS trials, only one of the commercial checks was among and not significantly different from the top fifteen hybrids (Table 6).

**Table 6:** BLUPs for the best 15 grain yield (GY) hybrids for all the trials and the MET analysis.

MET analysis		CS11-WS		CS11-WW		CS12-WS		CS12-WW	
Line	GY (ton/ha)	Line	GY (ton/ha)	Line	GY (ton/ha)	Line	GY (ton/ha)	Line	GY (ton/ha)
B14A	8.8*	NC408	3.3**	CML61	7.4****	NC334	9.9***	CML45	14.8****
CML115	8.7***	Mp04:97	3.3**	A&MY07-222	7.0***	A&MY07-222	9.6**	Mp707	13.5***
Tzi11	8.4***	CML108	3.2****	CML423	7.0****	CML-322R…	9.6**	CML264	12.8**
NC334	8.1****	CML423	3.2**	CML108	6.9****	CML77	9.5*	A&MY07-226	12.5****
CML264	8.0***	Mo47	3.2**	SC212M	6.7****	CML45	9.1**	CML52	12.3****
A&MY07–222	7.8****	NC260	3.2**	NC318	6.6****	[MBRC5BcF14…	9.0**	CML115	11.9***
NC370	7.8****	NC358	3.1**	DTPYc9-F46…	6.5****	CML254	9.0 ^NS^	Tzi11	11.7***
CML381	7.5****	DTPYC9-F134…	3.0*	CML264	6.4**	B14A	8.9 ^NS^	NC370	11.6****
Mp339	7.4*	DTPYC9-F143…	2.9*	LaPosta…F64…	6.4**	CML381	8.7*	SC357	11.4***
Check3	7.4****	CML348	2.9*	NC332	6.2**	M162W	8.6*	NC334	11.4***
CML45	7.3****	NC264	2.9*	LaPosta…F180…	6.2***	CML382	8.5*	[MBR-ET…	11.4***
Check4	7.2****	Mo45	2.7*	T234	6.2*	A&MY07_219	8.5*	Check3^a^	11.2****
CML261	7.2***	CML331	2.7*	Check2	6.2****	K148	8.5*	CML332	11.0***
LaPosta…F180…	7.0****	T234	2.7^NS^	Tzi8	6.1*	NC336	8.4 ^NS^	CML10^a^	10.9****
NC366	4.7****	A&M407–004	2.7 ^NS^	Check3^a^	6.1****	NC318 ^a^	8.3*	NC322^a^	10.8****
**Mean**	4.3	**Mean**	1.8	**Mean**	4.3	**Mean**	5.7	**Mean**	7.6

CS11-WS College Station 2011 water stress, CS11-WW College Station 2011 well watered, CS12-WS College Station 2012 water stress, and CS12-WW College Station 2012 well watered.

^a^ These testcross hybrids were significantly lower yielding than the top yielding hybrid in that environment.

The MET analysis was performed using Eq. (1) without spatial adjustment. For the rest of the analysis, the BLUPs for each individual trial were obtained using Eq. (2). Eq. (2) was expanded to include AR1 x AR1 terms for row and column spatial effects. The 15 best lines in each trial were mostly significantly different from the mean of the test but not from each other^a^; *, **, ***, **** indicate statistical significance at the 5%, 1%, 0.1%, and 0.01% level respectively, NS indicate non-significant differences.

For aflatoxin resistance, the best testcrosses (Table 7) were numerically better but generally not significantly different from most of the checks (data not shown) in every environment; this is likely because the tester selected, Tx714, has very high aflatoxin susceptibility, which was anticipated to identify inbreds with non-recessive forms of resistance [69]. However, aflatoxin values for many lines demonstrated an aflatoxin susceptibility statistically greater than the commercial checks; overall BLUPs from Eq. (1) ranged from 215 up to 1068 ng g^−1^. Testcrosses of the lines bred in tropical and sub-tropical areas generally exhibited decreased aflatoxin susceptibility when compared to testcrosses from temperate materials. These results indicate the presence of favourable alleles for stress tolerance and *Aspergillus* ear rot disease resistance in exotic material. The presence of favourable genetic variation in exotic germplasm has been previously reported by different authors, including for aflatoxin resistance [43,45–47,96].While the lowest accumulating testcross hybrids cannot be recommended per se, the best performing inbreds are expected to be useful in breeding for decreased aflatoxin susceptibility.

**Table 7:** BLUPs for the 15 lowest aflatoxin accumulating hybrids for all the trials and the MET analysis.

MET analysis		CS11-WW		CS12-WW		MS12-WW	
Line	Aflatoxin (ng g−1)	Line	Aflatoxin (ng g−1)	Line	Aflatoxin (ng g−1)	Line	Aflatoxin (ng g−1)
CML77	20**	CML77	87****	CML258	0^NS^	Mp705	22***
Tzi18	40**	Mp04:97	112****	A619	0^NS^	H95	23***
M37W	43***	Mp04:96	138****	B46	0^NS^	Mp07:121	25***
Mp04:97	43****	CML108	161****	C103	0^NS^	M37W	29**
NC340	47***	CML176	174***	CO125	0^NS^	NC290A	33**
KUI44	47*	Tzi9	177***	GT-AIRTP-W	0^NS^	NC342	34**
Tzi11	49^ns^	CML343	181***	A682	0^NS^	NC358	35**
CO125	51**	NC366	185***	KUI2007	0^NS^	DTPYc9-F46…	44**
CML52	51**	NC334	193*	Ki3	0^NS^	Mt42	46**
A619	53^ns^	CML258	195***	Tx772	0^NS^	Ki43	46**
CML342	55 ^ns^	CML342	197*	Tzi11	0^NS^	[MBR-…	49**
SC54	55 ^ns^	CML376	200*	Mo17	0^NS^	CML238	52*
CML264	56*	CML264	221*	CI187–2	0^NS^	LaPosta…F71…	53*
Mp07:121	58***	NC370	227*	NC290A	0^NS^	Check4	59****
CML381	58*	Tzi10	246**	CML5	0^NS^	CML52	60*
**Mean**	152	**Mean**	659	**Mean**	30	**Mean**	365

CS11-WW College Station 2011 well watered, CS12-WW College Station 2012 well watered, MS12-WW Mississippi state 2012 well watered. The MET analysis was performed using Eq. (1) on log10(aflatoxin + 10) transformed data without spatial adjustment, and back-transformed. The 15 best lines in each trial were mostly significantly different from the mean of the test but never from each other; *, **, ***, **** indicate statistical significance at the 5%, 1%, 0.1%, and 0.01% level respectively or, NS indicate non- significant differences.

### Genetic diversity, population structure and estimation of kinship matrix

Of the 400 lines, only 346 lines were able to successfully produce seed under conditions of the four Texas nurseries. The genetic diversity analysis between the 346 inbred lines indicated that the majority of the lines are quite distantly related to each other. Between all pairs of lines, the mean and median genetic distance was 0.68 and 0.69, respectively. Only 0.1% of the pairs of entries exhibited a genetic distance of less than 0.2, 2.7% of the entry pairs exhibited a genetic distance of less than 0.5, and 64% of the entry pairs exhibited a genetic distance less than 0.7 (S1 Fig.). These results suggested that the majority of the lines are equally distantly related in absolute number of shared alleles, however there were still patterns in allele sharing that resulted in observed population structuring. The population structure was determined using the software Structure [77,97], a preliminary analysis was performed to estimate the optimum K (number of populations) using the rate of change in the log probability, as measured by the ad hoc statistic ∆K. This analysis found that the optimum subpopulation number was K = 4 and these clusters roughly correspond to tropical, temperate, B73, and a mixed group. These results are similar to previous studies [49,67]. However, the Northern U.S./B14 stiff stalk lines clustered with non-stiff stalk lines (Mo17 related lines) [51], which is uncommon. This likely occurred because the Northern temperate germplasm is poorly represented in the current association panel. In contrast, the B73 group is small but well-defined.

**Fig 1:**
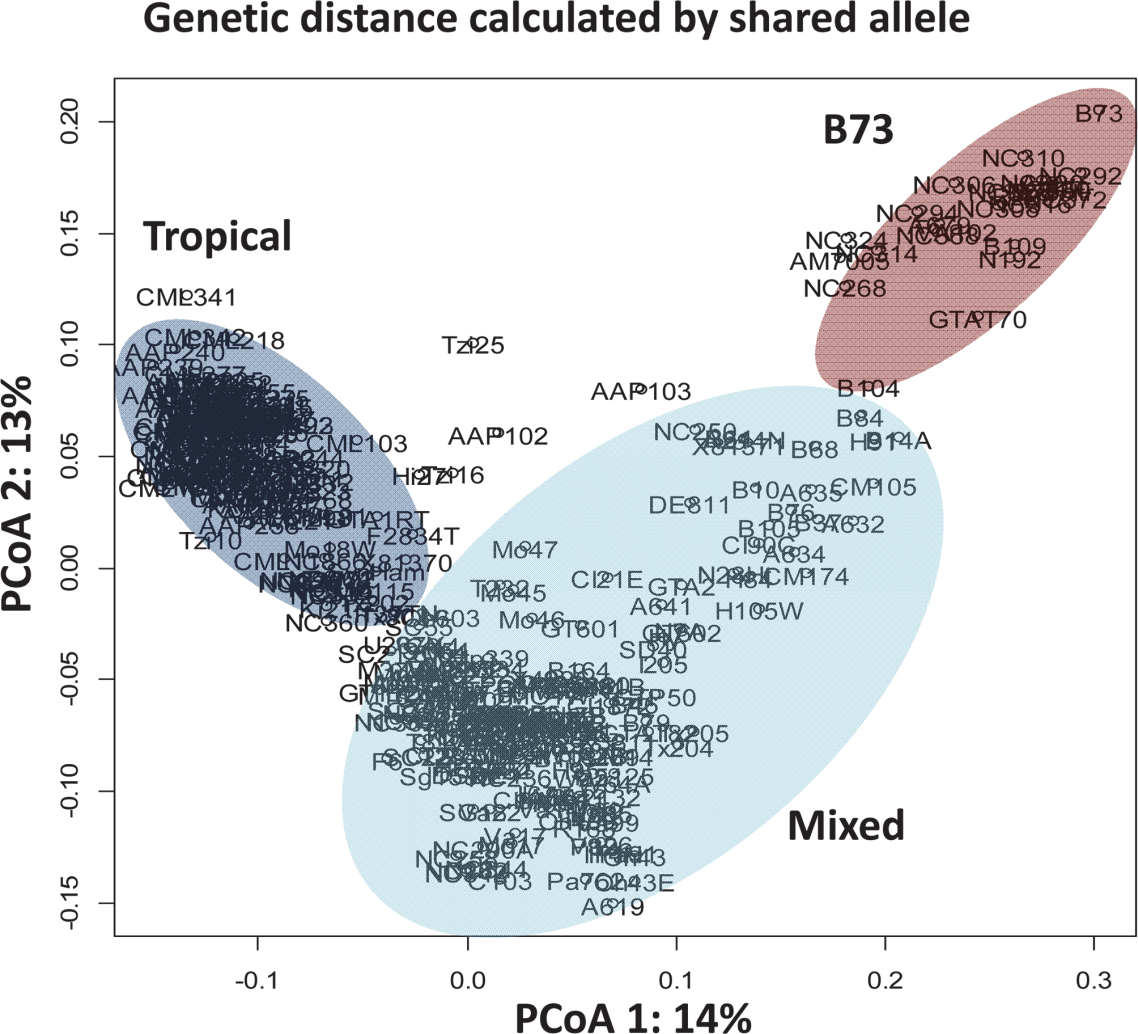
PCoA eigenvector plot of maize inbred lines that composed the diversity panel in this study. Nei’s (1972) genetic dissimilarly matrix was calculated from 1999 SNP markers.

The population structure results were similar to those visualized by the pairwise genetic distance matrix using a PCoA. The first two eigenvectors used to graph the data explained 14% and 13% of the variation, respectively, and separated three general clusters containing tropical lines, B73 types and a mixed group (Fig. 1). The mixed cluster is ill-defined and contains the non-stiff stalks and most southern US inbred lines. To explain 80% of the variation, over 100 PCoA eigenvectors were required, which further corroborated the weak structure between the inbred lines.

### GWAS analysis for grain yield and 500 kernel weight

**Fig 2:**
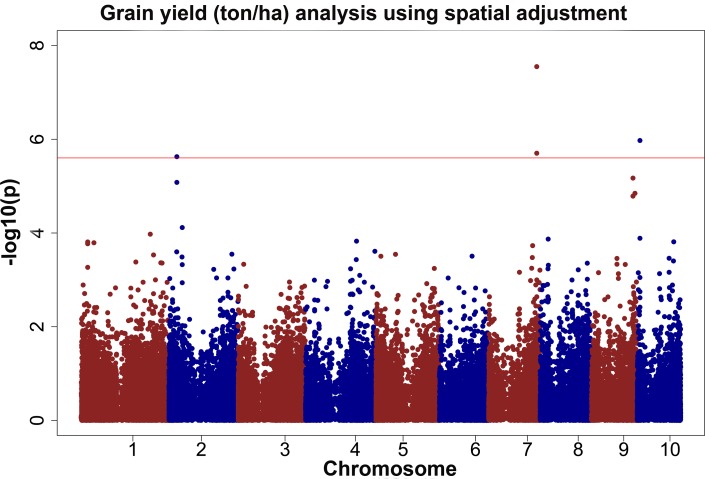
GWAS results for grain yield (ton/ha) using spatial adjustment. The phenotypic observation was the BLUPs for the MET analysis, which includes AR1 x AR1 terms to adjust for column and row effects. The red line represents the threshold value after correcting for multiple testing using the M_eff_ or FDR.

Based on the heritabilities shown in Table 2 for the different traits collected, it is clear that there was enough genetic variation in this study to perform a GWAS study to identify QTVs for most traits collected. For the GWAS analysis for grain yield four different analyses were performed using raw means, and the BLUPs extracted from equations 1 to 3 and the results were compared for consistency. Five QTVs for grain yield on chromosomes two, seven (two variants), nine (not shown in Fig. 2), and ten were identified across most analyses (Fig. 2). The allelic effects for the different QTV ranged from 0.14 to 0.59 ton/ha, and the amount of phenotypic variation explained ranged from 3 to 5% (Table 8).

**Table 8:** GWAS results for grain yield (ton/ha).

SNP	CHR	MAF	FDR_adjusted_P	Log10	Effect (ton/ha)	R^2^ (%)	QTV	APE
**CS11-WW-US^a^**								
S7_164955163	7	0.08	0.0003	8.25	0.37	4.9	QTV2	C
S9_142746374	9	0.26	0.0147	6.32	0.28	3.6	QTV3	G
S9_142746338	9	0.27	0.0197	6.02	0.28	3.4	QTV3	T
S2_27482479	2	0.22	0.0585	5.42	0.26	3	QTV1	C
**CS12-WS (average from raw data)**								
S2_27482431	2	0.24	0.0212	6.21	0.59	4.7	QTV1	A
S2_27482479	2	0.22	0.0212	6.16	0.59	4.7	QTV1	C
**CS12-WS-US^a^**								
S7_164955163	7	0.08	0.0005	8.11	0.42	5	QTV2	C
S9_142746374	9	0.26	0.0083	6.57	0.33	3.9	QTV3	G
S2_27482479	2	0.22	0.0133	6.08	0.31	3.6	QTV1	C
S9_142746338	9	0.27	0.0133	6	0.32	3.5	QTV3	T
S2_27482431	2	0.24	0.0133	5.97	0.31	3.5	QTV1	A
**CS12-WW-US^a^**								
S7_164955163	7	0.08	0.0028	7.35	0.14	4.9	QTV2	C
S2_27482431	2	0.24	0.0457	5.69	0.28	3.7	QTV1	A
S2_27482479	2	0.22	0.0457	5.63	0.28	3.6	QTV1	C
S9_142746374	9	0.26	0.0457	5.46	0.28	3.5	QTV3	G
S9_149545863	9	0.06	0.0457	5.43	0.37	3.5	QTV4	T
**MET analysis with spatial adjustment^b^**								
**SNP**	**CHR**	**MAF**	**FDR_adjusted_P**	**Log10**	**Effect**	**R^2^ (%)**	**QTV**	
S7_164955163	7	0.08	0.0017	7.6	0.35	4.5	QTV2	C
S10_10246117	10	0.24	0.0326	6	0.26	3.4	QTV5	G
S7_164954968	7	0.09	0.0361	5.7	0.32	3.2	QTV2	T
S2_27482479	2	0.22	0.0361	5.6	0.25	3.2	QTV1	C

^a^ The phenotypic observation used for the GWAS was the BLUP for the hybrid effect obtained using model in Eq. (3).

^b^ The phenotypic observation used for the GWAS was the BLUP for the hybrid effect obtained using the MET model in Eq. (2) including AR1 x AR1 terms for row and column effects.

Significant markers associated after correcting for multiple testing (SNP) using either FDR or M_eff_, their MAF, FDR adjusted p value of association, p value of the association [-log10 (p)], allele estimated effect, percentage of variation explained by marker (R^2^), the assigned name of the QTV in this study, and the allele of positive effect (APE) which increased yield.

QTV1, QTV2, and QTV3 were detected under WW and WS trials in 2011 and 2012, respectively (Table 8). These QTV were also detected in the MET analysis that adjusted for field spatial variation (Fig. 2). QTV1 had the strongest effect in the non-irrigated trial in 2012. No QTV were detected in the non-irrigated trial in 2011, likely due to low overall heritability. QTV4 was only significant in the well-irrigated trial in 2012 and QTV5 was only significant in the MET analysis that adjusted for field spatial variation (Table 8). Several linkage mapping studies have reported multiple QTLs for grain yield, but to our knowledge this has not yet been examined using association approaches or in testcrosses [51,98–105]. These previous studies based on linkage mapping identified one to five linkage QTLs for grain yield, explaining 20 to 35% of phenotypic variation for all QTLs together.

QTV1 found in this study for grain yield is located in bin 2.03, and the exact SNP location can be found within the locus name in Table 9. No QTLs associated with grain yield have been reported in this bin by other authors and this SNP is in the *abph1*—aberrant phyllotaxy1- gene (Maize B73 RefGen_v2available at www.maizegdb.com/). Gene *abph1* is expressed in the shoot apical meristems, and mutations in this gene alters the regular arrangement of leaves and flowers [106]. Despite LD decaying rapidly in this area of the genome, further investigation must be done to confirm that this SNP is only associated with the *abph1* locus, and not other linked genes.

**Table 9:** Summary of the most promising QTV variants found in this study.

SNP	QTV variant	Bin	Chr.	Allele 1	Allele 2	Description	Plausible transcript
**Grain yield (ton/ha)**							
S2_27482431	QTV1	2.03	2	A* ^b^	C	PUT-2–171a-Zea_mays-13770	GRMZM2G035688
S7_164955163	QTV2	7.04	7	A	C* ^b^	Protein unknown function	GRMZM2G009320
S9_142746374	QTV3	9.06	9	A	G* ^b^	Clp amino terminal domain	GRMZM2G150598
S9_149545863	QTV4	9.07	9	C	T* ^b^	unknown motif	GRMZM5G864133
S10_10246117	QTV5	10.02	10	G* ^b^	T	unknown motif	GRMZM2G475197
**Plant height (cm)**							
S7_164955163	QTV2	7.04	7	A	C* ^b^	Protein unknown function	GRMZM2G009320
S3_168307280	QTV6	3.05	3	A* ^c^	C	Chromatin assembly factor I	GRMZM2G096458
**Ear height (cm)**							
S2_34433893	QTV7	2.04	2	C	T* ^c^	Proton-dependent oligopeptide transporter	GRMZM2G138731
S4_62573339	NS^a^	4.05	4	C*	G^c^	four cysteine-rich zinc finger protein	GRMZM2G153722
S4_173817044	NS^a^	4.07	4	C	T* ^b^	Promoter region	GRMZM2G549279
S4_173996901	NS^a^	4.07	4	A	G* ^b^	Promoter region	GRMZM2G010755
**Days to anthesis or days to silk**							
S7_164955163	QTV2	7.04	7	A	C* ^b^	Protein unknown function	GRMZM2G009320
S8_131176630	QTV8	8.05	8	C* ^b^	T	Protein tyrosine kinase	GRMZM2G120839
S4_173817044	NS^a^	4.07	4	C	T* ^b^		GRMZM2G549279
S8_123509373	QTV9	8.05	8	C	G* ^b^	Protein unknown function	GRMZM2G479987
S3_1775697	QTV10	3.02	3	A	C*	Epsin N-terminal homology (Domain)	GRMZM2G123499

*Allele with the positive effect

^a^These QTV were just under the significance threshold after multiple test correction but were consistent skyscrapers observed in multiple tests.

^b^ This is the allele in both Tx714 and B73 based on citation [117]

^c^ This is the allele in B73 but Tx714 is unknown based on citation [117].

SNP position test, QTV name, bin, chromosome, SNP_1 allele one present, SNP_2 allele two, description of the translated protein motif, plausible transcript as reported in the B73 genome sequence. The SNP name is also the position on the maize genome using assembly AGPv2.

**Fig 3:**
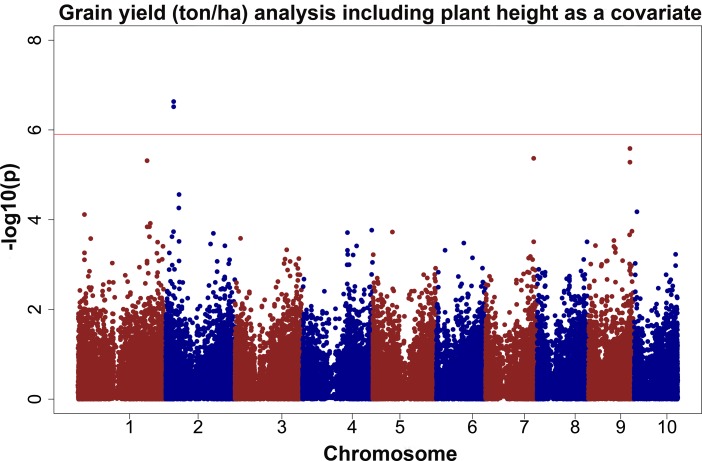
GWAS results for grain yield (ton/ha) using spatial adjustment and a covariate for plant height. The phenotypic observation was the BLUPs for the MET analysis, which includes AR1 x AR1 terms to adjust for column and row effects. In addition, this model includes a centered and standardized covariance for plant height. The red line represents the threshold value after correcting for multiple testing using the M_eff_ or FDR.

QTV2 is located in bin 7.04, the allelic effect ranged from 0.14 to 0.42, and the percentage of explained phenotypic variation ranged from 4.5 to 5% (Table 8). In addition to yield, QTV2 was also detected as associated with plant height, days to anthesis and days to silk, suggesting a pleiotropic effect on multiple traits (Table 9). In a recent meta-analysis of Texas commercial yield trial data, the R^2^ of plant height and grain yield was determined to be 0.61, indicating the importance of robust tall plants under Southern stressed growing conditions and further supporting the possibility of pleiotropy of this locus [14]. In order to further address this question, an association analysis was performed including plant height as a covariate in Eq. (1) and (2). The QTV2 variant was no longer significant for grain yield, providing an additional line of evidence that this QTV affects grain yield via a positive pleiotropic effect with height and may be enhancing overall plant vigor (Fig. 3). Austin and Lee [101] found a QTL for grain yield in the same bin that explained 3.9% of the phenotypic variation; however, their sparse map makes it difficult to confirm co-location with QTV2. Schön et al. [107] using 1000 F_4:5_ maize testcross progeny developed by Pioneer Hi-Bred in 1995, identified a QTL in the same chromosome bin, which was detected across seven environments in the Corn Belt.

To identify potential candidate genes for QTV2, LD was investigated and found to decay within 0.2 kilobases (kb) upstream and showed no LD with the closest marker 225 (kb) downstream (data not shown). Different authors have reported that LD decays rapidly in maize around 1 to 10 kb; specifically for chromosome seven, linkage disequilibrium has varied from 2 to 5 kb depending on the region and the germplasm [52,108–111]. Assuming that the LD follows the same pattern observed for the upstream area from QTV2, a plausible transcript corresponds to a protein with an unknown function (Table 9) that is expressed in vegetative stage 1 (V1) (Maize eFP browser) [112,113].

QTV3 is located in bin 9.06, the allelic effect ranged from 0.28 to 0.33 ton/ha across environments, and the percentage of explained phenotypic variation ranged from 3.5 to 3.9% (Table 8). No QTLs have been reported in bin 9.06 for grain yield to our knowledge. LD near the QTV3 variant is higher than average, which is consistent to previous reports for chromosome nine [52,110,111]. There are six transcripts upstream from QTV3 within a high level of LD. Downstream from the QTV3 variant, there are two additional transcripts (GRMZM2G150594 GRMZM2G573775) in LD with the variant. Additional work will be needed to narrow down which of the seven possible transcripts (if any) are responsible for the positive association with grain yield. QTV4 (bin 9.07), and QTL5 (bin 10.02) are in locations that have not been previously reported to contain grain yield QTL, and correspond to genes of unknown function (Table 8).

This study did not detect a significant QTV for 500-kernel weight after stringent corrections for multiple testing. However, a peak near significance was consistently observed on bin 9.01 across different trials and both of the MET analysis (data not shown). The effect estimates ranged from 2.3 to 3.6 g per 500 kernels, and explained 3.8 to 4.3% of the phenotypic variation. Although this peak was not significant after correcting for multiple testing using FDR and M_eff_, this SNP warrants further investigation based on consistency and the fact that other authors have reported a linkage QTL for 300 kernel weight, named q300k21, in the same bin [114–116]. Correction for multiple testing can be too stringent, causing false negatives and loss of information.

The effect sizes of significant QTV for yield, which ranged from 3 to 5%, appeared larger in magnitude than might be expected for a complex quantitative trait; we believe there are a few potential causes. First, heterosis from the tester would be expected to mask phenotypic variation at many alleles. For all QTVs the tester (Tx714) has the allele of positive effect [117], suggesting theseQTVs show either additive effects or dominant deleterious effects. Second, with such a diverse panel, more extreme deleterious alleles would be expected to be present than in a narrow elite panel. Indeed, we find that the alleles with the positive effect are more common in the population and examining the QTVs in ex-PVP lines [45], only a few have the alleles of negative effect; these are generally in the oldest lines suggesting they have been selected out of more elite maize [117]. Third, given the moderate population size it is reasonable to expect the ‘Beavis effect’ or ‘winners curse’ to be present in this study and all allelic effects may be overestimated. Fourth, it is possible that population structure is not fully accounted for [64]; however, this seems less probable after examining the diverse origins of the individuals with these SNPs.

### GWAS for plant and ear height

**Fig 4:**
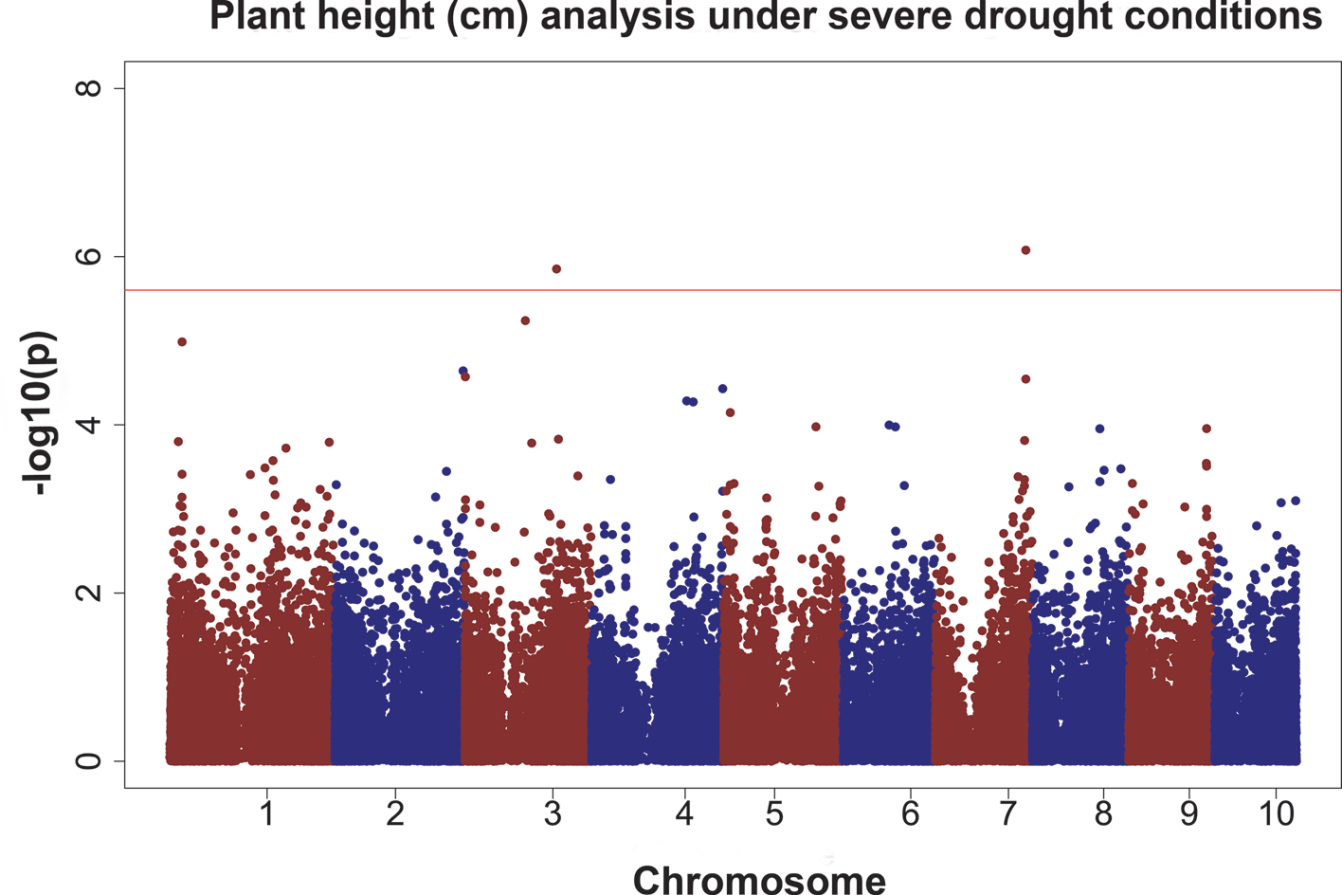
GWAS results for plant height (cm). The phenotypic observation was the BLUPs for the CS11-DTYL trial. The red line represents the threshold value after correcting for multiple testing using the M_eff_ or FDR.

Two QTVs (QTV2 and QTV6) were detected for plant height (Fig. 4). QTV2 was previously described for grain yield (Table 8 and 9). Several studies have reported a QTL for height at bin 7.04 [104,105,107]. The effect of QTV2 ranged from 5.3 to 5.6 centimetres, and explained 4.6 to 5% of the phenotypic variation (Table 10). The other SNP, QTV6 in bin 3.05, had an effect that ranged from 3 to 3.2 centimetres and explained 4.7 to 4.8% of the phenotypic variation (Table 10). A QTL for plant height has been reported previously in bin 3.05 using a bi-parental cross between lines Ki3 and CML139 [118]. Both QTV2 and QTV6 were detected in the non-irrigated and in the irrigated trials in 2011 (Fig. 4).

**Table 10:** GWAS results for plant and ear height (cm).

SNP	CHR	MAF	FDR adjusted P	Log10	Effect (cm)	R^2^ (%)	QTV	APE
**Plant height**								
**CS11-WS-US^a^**								
S7_164955163	7	0.08	0.0429	6.08	5.3	5	QTV2	C
S3_168307280	3	0.36	0.0429	5.85	3	4.8	QTV6	A
**CS11-WW-US^a^**								
S3_168307280	3	0.36	0.0577	5.81	3.2	4.7	QTV6	A
S7_164955163	7	0.08	0.0577	5.73	5.6	4.6	QTV2	C
**Ear height**								
**CS12-WS (average from raw data)**								
S2_34433893	2	0.31	0.0391	6.2	3.9	6.3	QTV7	T
**CS12-WS-US^a^**								
S4_173996901	4	0.13	0.0811	5.88	3.8	4.6	NS^C^	G
SNP	CHR	MAF	FDR adjusted P	Log10	Effect	R^2^ (%)	QTV	
**CS12-WW (average from raw data)**								
S4_173817044	4	0.14	0.0789	5.69	6.5	5	NS^C^	T
S4_62573339	4	0.41	0.0789	5.59	4.4	5	NS^C^	C
**CS12-WW-US^a^**								
S4_173996901	4	0.13	0.1468	5.62	4.3	4.4	NS^C^	G
**RCBD^b^**								
S4_173817044	4	0.13	0.362	5.23	3.8	4.2	NS^C^	T

^a^ The phenotypic observation used for the GWAS was the BLUP for the hybrid effect obtained using model in Eq. (3).

^b^ The phenotypic observation used for the GWAS was the BLUP for the hybrid effect from the MET model in Eq. (1).

^C^These QTV were just under the significance threshold after multiple test correction but were consistent skyscrapers observed in multiple tests.

Significant markers associated after correcting for multiple testing (SNP), their MAF, FDR adjusted p value of association, p value of the association (-log10 (p)), allele estimated effect, percentage of variation explained by marker (R^2^), the assigned name of the QTV in this study, and the allele of positive effect (APE) which increased height.

**Fig 5:**
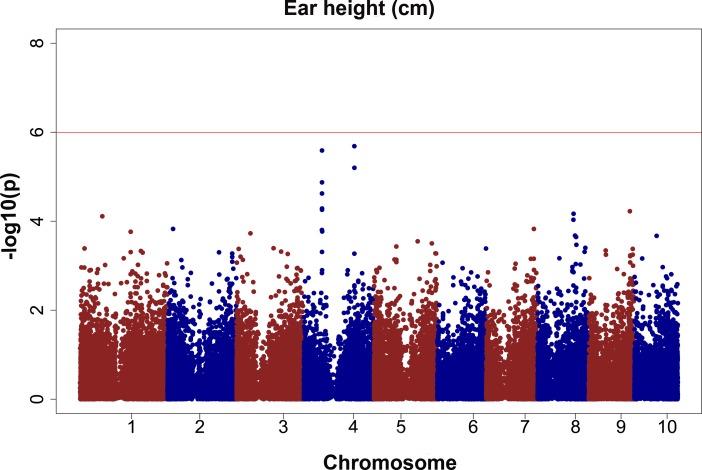
GWAS results for ear height (cm). The phenotypic observation was the BLUPs for the CS12-WW trial.

For ear height, only one significant SNP, QTV7 (bin 2.04) was detected after correcting for multiple testing and only in the non-irrigated trial in 2012 (Table 10). It had an estimated effect of 3.9 centimetres and explained is 6.3% of the phenotypic variation. No QTL have been previously reported for ear height in this bin to our knowledge. Three additional peaks were consistently detected across different analyses, and the effects were strongest in the CS12-WW trial (Fig. 5). These SNPs are worthy of additional consideration despite the fact that none of them were significant after adjusting for multiple testing. The SNP S4_62573339 is located in bin 4.05, had an allele effect of 4.4 centimetres, and explained 5% of the phenotypic variation (Table 10). The SNP falls within a putative transcript; however, the LD extends around 100 kb in this area, indicating that this transcript may not be the causal gene. The strong LD in this area may indicate that this region of the genome has been under recent selection. Different authors have reported that LD generally decays around 1 to 5 kb for chromosome four [52,108–111]. A QTL for plant height, but not ear height, has been previously reported in bin 4.05 by different authors [102–105].

Significant peaks associated with SNPs S4_173817044 and S4_173996901 are located in bin 4.07. The effect of the S4_173817044 SNP ranged from 3.8 to 6.5 centimetres, and the percentage of explained phenotypic variation ranged from 4.2 to 5%. The effect of the S4_173996901 SNP ranged from 3.8 to 4.3 centimetres. The amount of phenotypic variation ranged from 4.4 to 4.6%. Similar to the previous QTL for ear height, neither SNP falls within a bin where a QTL has been reported by other authors. Additionally, the LD decay around 100 bp in this area of chromosome four and based on the Maize B73 RefGen_v2 genome, these SNPs seems to be in the promoter regions of different uncharacterized transcripts (Table 9).

### GWAS for flowering time traits

**Fig 6:**
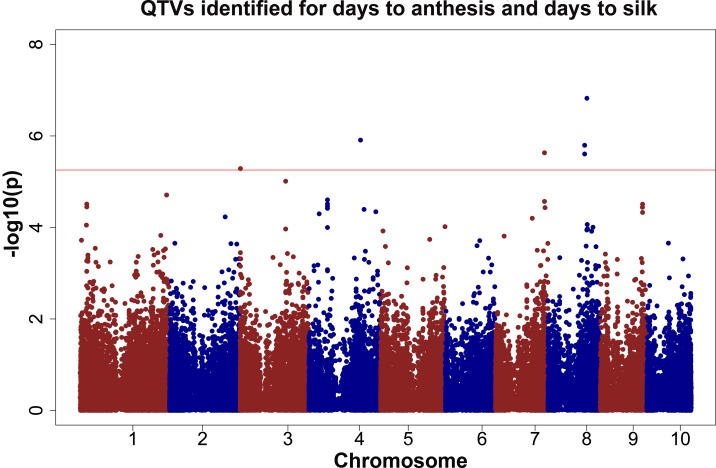
GWAS results for days to anthesis and days to silk. The phenotypic observation was the BLUPs for the CS12-WW trial. The red line represents the threshold value after correcting for multiple testing using the M_eff_ or FDR.

For the days to silk and days to anthesis traits, only one GWAS analysis was performed using the entry mean as the phenotype observation. Multiple QTVs were found (Fig. 6) with effects ranging from 0.5 to 1.8 days and the percentage of explained phenotypic variation ranging from 4.2 to 7.4% (Table 11). QTV2, previously reported for grain yield and plant height, was also detected for days to anthesis and days to silk in the well-watered CS11-WW and CS12-WW trials. Buckler et al. [119] reported three QTLs for days to anthesis (PZA03624, PZA03728, PZA-1744) and four QTLs for days to silk (PHM15501.9, PZA00986.1, PZA02722.1, PZA01044.1) on chromosome seven. However, based on the physical location and genetic distance none of the markers are located in bin 7.04.

**Table 11:** GWAS results for days to anthesis and days to silk.

SNP	CHR	MAF	FDR_adjusted_P	Log10	Effect (days)	R2 (%)	QTV	APE
**Days to anthesis**								
**CS12-WS^a^**								
S7_164955163	7	0.07	0.0006	8.03	1.8	7.4	QTV2	C
**CS12-WW^a^**								
S8_131176630	8	0.26	0.0088	6.85	0.9	5.8	QTV8	C
S4_173817044	4	0.14	0.0314	5.91	0.8	4.9	NS^a^	T
S8_123509373	8	0.37	0.0314	5.78	0.6	4.7	QTV9	G
S7_164955163	7	0.08	0.0314	5.65	1.8	4.6	QTV2	C
S8_123511933	8	0.38	0.0314	5.59	0.5	4.6	QTV9	G
S3_1775697	3	0.41	0.0571	5.25	0.6	4.2	QTV10	C
**RCBD^a^**								
S7_164955163	7	0.07	0.0026	7.37	1.6	6.4	QTV2	C
S8_131176630	8	0.27	0.0563	5.74	0.9	4.6	QTV8	C
S4_173817044	4	0.14	0.0563	5.74	1.1	4.6	NS^a^	T
**Days to silk**								
**CS12-WS^a^**								
S7_164955163	7	0.07	0.0006	8.01	1.8	7.4	QTV2	C
**CS12-WW^a^**								
S8_131176630	8	0.26	0.0092	6.82	-1	5.8	QTV8	C
S4_173817044	4	0.14	0.0304	5.91	1.2	4.9	NS^a^	T
S8_123509373	8	0.37	0.0304	5.8	0.8	4.8	QTV9	G
S7_164955163	7	0.08	0.0304	5.63	1.3	4.6	QTV2	C
S8_123511933	8	0.38	0.0304	5.61	0.8	4.6	QTV9	G
S3_1775697	3	0.41	0.0529	5.29	0.8	4.3	QTV10	C
**RCBD^a^**								
S7_164955163	7	0.07	0.0029	7.33	1.6	6.4	QTV2	C

^a^Phenotypic observation for the GWAS was the average for days to anthesis or days to silk

Significant markers associated after correcting for multiple testing (SNP), their MAF, FDR adjusted p value of association, p value of the association [-log10 (p)], allele estimated effect, percentage of variation explained by marker (R^2^), the assigned name of the QTV in this study, and the allele of positive effect (APE) which increased days to flowering.

QTV8 (bin 8.05) was detected for both days to anthesis (effect of 0.9 days) and days to silk (effect of 1 day), explaining 5.8% of the phenotypic variation for both traits. Two different SNPs were found for QTV9 (bin 8.05), S8_123509373 and S8_123511933, separated by 2.5 kb. The effect for these QTL variants ranges from 0.5 to 0.8 and the phenotypic variation ranges from 4.6 to 4.7% (Table 11). Buckler et al. (2009) reported three QTLs for days to anthesis (PZA00908.2, PZB02155.1, PZA00675.1) and three QTLs for days to silk (PHM4711.14, PZB02155.1, PHM1834.47) on this chromosome across eight environments in the NAM panel. One of the QTL reported is located on chromosome eight in bin 8.05 (locus pzb02155), located between position 123,542,426 and 125,974,265, which is 30 kb downstream from QTV9. LD extends for 3 kb upstream from QTV9 (results not shown); however, downstream from the QTV9, there is gap between the markers of 80 kb. As a consequence, this study cannot definitively determine if QTV9 and locus PBZ02155.1 are the same QTV or not. Finally, QTV10 had an effect of 0.6 for days to anthesis and 0.8 for days to silk, and explaining 4.2% of the phenotypic variation for days to anthesis and 4.3% for days to silk (Table 11).

This study did not find any significant QTL variants for ASI despite multiple analyses run using the average of the raw data, and the MET analysis described in Eq. (1) and (2). Buckler et al.’s [119] study was the most powerful to date for flowering time QTV detection, and many but not all of the QTV were novel to a particular study. This lack of co-localization raises the question of whether the differences observed were because of the use of different germplasm, different environments, or the use of testcross hybrids. If germplasm caused the observed differences, this suggests that the hypothesis of multiple variants for common genes may play more of a role in temperate than in tropical germplasm. If different environments were the cause, this suggests that local testing is critical for relevance in association mapping. If the use of testcross hybrids were the cause, this suggests that for the most relevance to crop improvement, only testcross hybrids and a relevant tester should be used in GWAS studies conducted in crops grown as hybrids. It seems likely that the true cause is partially due to all these factors and their interactions.

### GWAS for aflatoxin resistance

**Fig 7:**
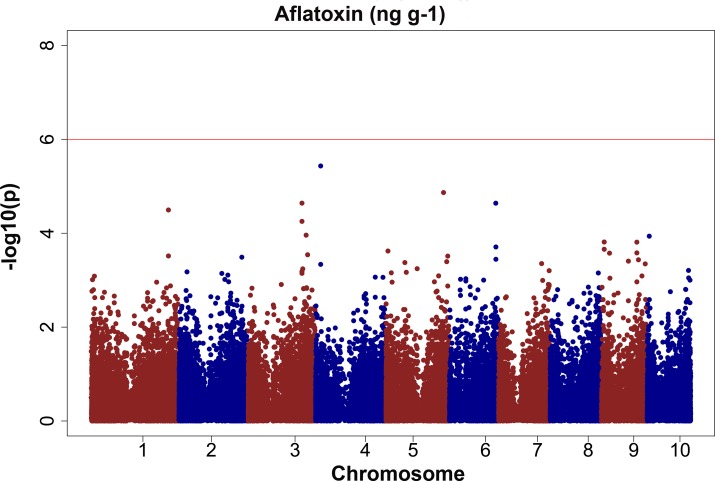
GWAS results for aflatoxin (ng g−1). The phenotypic observation was the BLUPs for the CS12-WW trial.

The GWAS analysis did not find any significant QTLs for the transformed aflatoxin data after correcting for multiple testing (Fig. 7) despite moderately high heritability and significant variation between the experimental hybrids. Three peaks appeared consistently for the irrigated trials CS11-WW and CS12-WW, and the combined MET analysis (Table 12). SNP: S3_185272026 (bin 3.06) had an allele effect of 9.4 ng g−1 and explained 6.06% of phenotypic variation (Table 12). This SNP corresponds to transcript GRMZM2G399433, which is highly expressed in the pericarp, embryo and endosperm, the silks and the cob during flowering and post-flowering [112]. These tissues are directly relevant for the infection of *A*. *flavus* to proceed under non-wounding inoculation. A QTL for aflatoxin and/or *Aspergillus* ear rot resistance has been previously reported in bin 3.06 [34,36,39]. This QTV did not have a significant effect on any of the other traits measured (yield, height, flowering time, etc.) indicating that the effect of this QTV is directly on aflatoxin or ear rot resistance, not via a co-varying trait.

**Table 12:** GWAS results for aflatoxin level.

SNP	CHR	MAF	FDR_adjusted_P	Log10	Effect	R2 (%)	APE	ANE
**CS11-WW^a^ Aflatoxin (ng g−1)**								
S4_17376432	4	0.32	0.2	5.48	9.1	5.27	C	G
S5_197707198	5	0.15	0.24	5.1	8.9	4.85	T	C
**CS12-WW^a^ Aflatoxin (ng g−1)**								
S3_185272026	3	0.3	0.34	5.11	9.4	6.06	A	G
**CS12-WW^a^ Aflatoxin (ng g−1)**								
S4_17376432	4	0.32	0.23	5.43	9.1	5.69	C	G
**RCBD Aflatoxin (ng g−1)**								
S5_197707198	5	0.15	0.27	5.22	8.9	4.98	T	C
S4_17376432	4	0.32	0.27	5.05	9.1	4.79	C	G

^a^ The phenotypic observation used for the GWAS was the BLUP for the hybrid effect obtained using model in Eq. (3) on (log10 [aflatoxin + 10]).

Plausible significant markers associated with (log10 [aflatoxin + 10]) after correcting for multiple testing (SNP), their MAF, FDR adjusted p value of association, p value of the association [-log10 (p)], allele estimated effect after back-transformation to (ng g−1), the percentage of variation explained by marker (R^2^), and the allele of positive effect (APE) which less desirably increased aflatoxin, and the desirable allele of negative effect (ANE).

The SNP: S4_17376432 (bin 4.03) had allele effects of 9.1 ng g−1 and explained phenotypic variation ranging from 5.3 to 5.7%. The transcript GRMZM2G013546 corresponds to this SNP marker and is highly expressed in the pericarp and husk of maize [36,41,112], and a strong QTL for aflatoxin resistance coming from the resistant line Mp313E has been found in this bin location by multiple authors [5,35]. Further evidence of the presence of a significant QTL in this bin comes from a recent meta-analysis study [120]. After building a consensus map, the study found linked markers extending from bins 4.02 to 4.04, which seems to contain six QTL for the three diseases presented, including aflatoxin and *Aspergillus* ear rot. Multiple QTL for ear rot resistance have been reported in bin 4.03 for resistance to ear rot diseases such as *Fusarium* and *Gibberella* [36,41]. The most likely reason that this QTV was not declared statistically significant in our study is that only a fraction of the lines in the panel have the allele, thus reducing our statistical power. Another possible explanation is the low marker density, which may not have been close enough to detect the causal polymorphism. The peak at SNP: S5_197707198 (bin 5.06) corresponds to the transcript GRMZM2G057789, which is highly expressed in the silks during the R1 stage [112]. Xiang et al. [36] reported a QTL responsible for ear rot resistance in the same bin 5.06. One reason for the relatively poor detection of QTV for aflatoxin could be the choice of a susceptible tester, allowing only dominant sources of resistance to be found. However it is dominant sources of resistance that would be of most use in commercial breeding, since a recessive source would require improving lines in two heterotic groups.

### Major findings of this study

Association mapping using candidate genes or at a whole genome level is now routine in many plant species [64,66,121–126] including maize. These studies have reported on average fewer associations than linkage mapping based studies, even for genes with large phenotypic effects. Although many maize association mapping studies have been conducted [127–129], there have been few previous reports of mapping in a hybrid testcross background which allows dominant alleles to be detected. This study found 10 QTV and other potential associations which were nearly significant after correcting for ∼50,000 tests; several of these QTVs co-located with QTL reported by others, which suggests that association mapping in a diverse set of germplasm using testcrosses is consistent and relevant. For QTV co-located to QTL reported from linkage mapping, this study identifies potential candidate genes, improves genetic resolution and provides independent confirmation of these loci. QTV novel to this study might not have been segregating in previous linkage mapping populations, and detection of these is a benefit of GWAS studies. Further investigation of these novel QTV will rule out possible false positives and validate their use for maize improvement. Detection power of association mapping is affected by several factors including sample size, population structure, the extent of LD, the magnitude of the phenotypic effect, and the quality and density of the SNP markers used [52,60,110,130]. The results here clearly highlight the importance of larger sample size to be able to detect associations with rare SNPs and genes of small effect, likely the most important genetic basis for complex traits such as drought tolerance and aflatoxin resistance. Yan et al. [52] reported that using a population of 500 individuals in GWAS can detect associations that explain as little as 3% of the phenotypic variation. Increasing the sample size to 1500 genotypes can detect associations that explain 1% of the phenotypic variation, but would not be practical given the resources needed for field analyses with replication. Similar trends have been obtained for QTL mapping, where it has been shown that increasing the number of individuals is more efficient than increasing the number of replications [107]. The importance of large sample size was highlighted by our results obtained for aflatoxin GWAS, where no significant associations were detected after correcting for multiple testing. In the case of maize we feel it best to err slightly towards type I error since it is relatively straight forward to confirm or refute candidates in subsequent studies. Therefore, we used two different methods of multiple testing corrections, with different advantages and disadvantages; improved methods with less type II error for crop species would be desired and is an ongoing area of research [61,83].

## Conclusions

This study used a diverse maize association mapping panel to identify genomic regions associated with grain yield, aflatoxin resistance and important agronomic traits in Southern US environments. Useful variation in diverse germplasm for aflatoxin resistance and drought tolerance was identified. Additionally, this diverse germplasm, when testcrossed to an elite, although older, Texas version of B73, has the potential to out-yield commercial hybrids sold in Texas but bred in the best Midwestern environments. This study found 10 QTVs for grain yield, plant and ear height, days to anthesis and days to silk with some co-localizing to previously reported linkage QTL, while others were novel, demonstrating the utility of GWAS to resolve and discover useful variation. Comparing different statistical adjustment models was useful to maximize the power of the data and to detect consistent QTVs. Once these QTVs are validated, they will be useful for molecular improvement of Southern maize germplasm and, if cloning is pursued, for understanding the basic biology of improvement of these traits.

## Supporting Information

S1 Fig Heatmap of genetic distances between lines. The pairwise genetic distances between lines according to Nei’s 1972 genetic distance were plotted in a heat map. The distribution of pairwise distance values in the upper left also shows the color legend. In the main figure, the dendrogram and relatedness are shown on the top and left. The names of the lines are shown on the right and bottom of the main figure.
(PDF)

S1 Table Raw phenotypic data. The raw phenotypic data is provided to allow genetic mapping using this panel with SNPs discovered in the future. Column headings are as follows: Rows—The number of rows in a plot; Row Width—the Row width in centimeters; Range Length—the plot length in meters; Loc—the location of the test; Year—the year of the test; Trial:$9—a unique combination of location, year, and treatment; Trial:$92—a unique combination of location, year, and test; Pedigre:$160.—the pedigree of the testcross hybrid inbred lines which were crossed to Tx714; Common name—another version of the inbred pedigree; Entry:$ a coded entry for analysis; Entry_No—the entry number; Panel:$15.—a stock number for the inbred; Inbredtag:$15.—a stock number for the testcross hybrid seed that was planted; Tagtrial:$15.- the unique barcode/ plot location in the field; Lox:$—the knocked out lipoxygenase gene in the Tx714 isogenic tester (must be either lox-4 or lox-5); Plot:$—the plot number, only unique within a trial; Row—the range in the field, only unique within a field location; Column—the row in the field, only unique within a field location; Pos—an identifier of row and column only unique within a field location; Rep—the replication only unique within a trial; DTA—days from planting to anthesis; DTS—days from planting to silk; ASI—anthesis silking interval; PH—plant height in centimeters; EH—ear height in centimeters; STD—the stand count in number of plants; PlotWt_gr—the total plot weight from the combine and hand harvest in grams; Moist—the percentage of moisture in the grain; kernel500Wt—the weight of 500 kernels in grams; Yield_plot—the yield in kilograms per hectare; Yield_BuAc—the yield in bushels per acre; PlotWt_Combine_gr—the plot weight from the combine in grams; Wt_Shelled_grs—the weight from hand shelling hand-harvested ears in grams; EarCount—the number of ears hand-harvested and hand shelled; poln_per—the percentage of pollinated kernels on hand-harvested ears; abortion_per—the percentage of kernel abortion on the ears; flav_per—a visual rating of the amount of *Aspergillus flavus* sporulation on the ears; NoKerRow1, NoKerRow2, NoKerRow3—the number of kernel rows on each of three randomly selected hand harvested ears; type:$—a visual rating of the kernel type (flint or dent); the amount of Aflatoxin in ng g−1; Log(Aflatoxin+10)—the log of aflatoxin in ng g−1 plus 10.
(XLSX)
