# Supplementary figures and images for: Genome Wide Association Study for Drought, Aflatoxin Resistance, and Important Agronomic Traits of Maize Hybrids in the Sub-Tropics
Source: PLoS One. 2015 Feb 25;10(2):e0117737. doi: 10.1371/journal.pone.0117737 (PMC4340625; doi:10.1371/journal.pone.0117737)

Color Key  
and Histogram

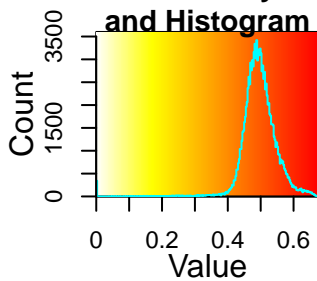

# Heatmap Genetic Distances

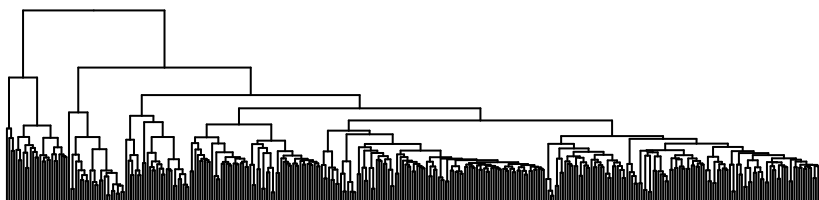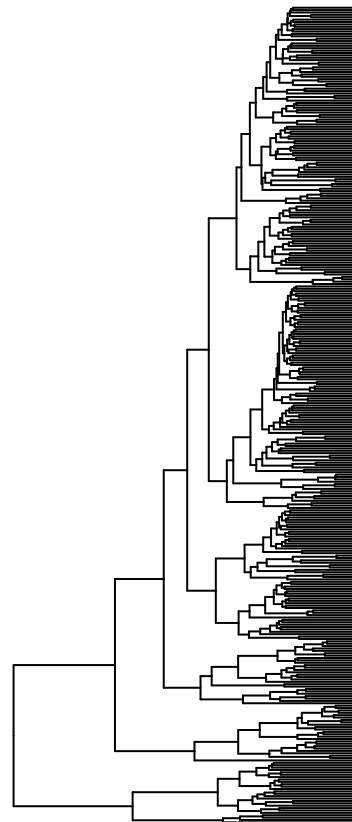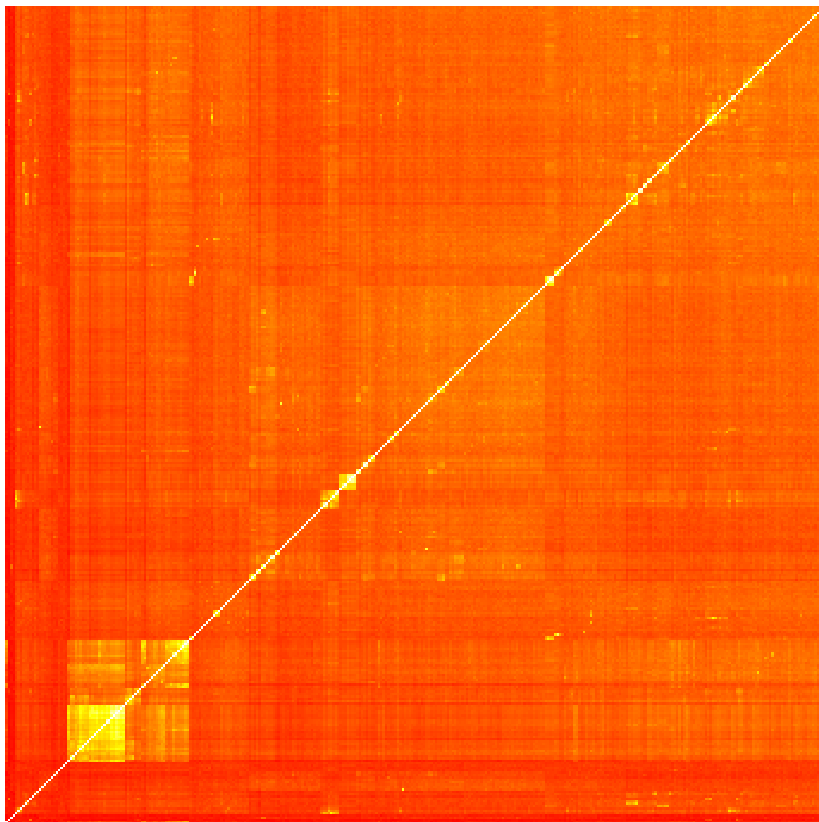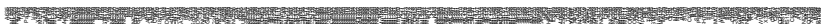

Supplement: S1 Fig — The pairwise genetic distances between lines according to Nei’s 1972 genetic distance were plotted in a heat map. The distribution of pairwise distance values in the upper left also shows the color legend. In the main figure, the dendrogram and relatedness are shown on the top and left. The names of the lines are shown on the right and bottom of the main figure. (PDF) [file pone.0117737.s001.pdf]
